# Refining SARS-CoV-2 intra-host variation by leveraging large-scale sequencing data

**DOI:** 10.1093/nargab/lqae145

**Published:** 2024-11-12

**Authors:** Fatima Mostefai, Jean-Christophe Grenier, Raphaël Poujol, Julie Hussin

**Affiliations:** Département de Biochimie et de Médecine Moléculaire, Université de Montréal, Québec, Canada; Research Center, Montreal Heart Institute, Québec, Canada; Mila - Quebec AI Institute, Université de Montréal, Québec, Canada; Research Center, Montreal Heart Institute, Québec, Canada; Research Center, Montreal Heart Institute, Québec, Canada; Département de Biochimie et de Médecine Moléculaire, Université de Montréal, Québec, Canada; Research Center, Montreal Heart Institute, Québec, Canada; Mila - Quebec AI Institute, Université de Montréal, Québec, Canada; Département de Médecine, Université de Montréal, Québec, Canada

## Abstract

Understanding viral genome evolution during host infection is crucial for grasping viral diversity and evolution. Analyzing intra-host single nucleotide variants (iSNVs) offers insights into new lineage emergence, which is important for predicting and mitigating future viral threats. Despite next-generation sequencing’s potential, challenges persist, notably sequencing artifacts leading to false iSNVs. We developed a workflow to enhance iSNV detection in large NGS libraries, using over 130 000 SARS-CoV-2 libraries to distinguish mutations from errors. Our approach integrates bioinformatics protocols, stringent quality control, and dimensionality reduction to tackle batch effects and improve mutation detection reliability. Additionally, we pioneer the application of the PHATE visualization approach to genomic data and introduce a methodology that quantifies how related groups of data points are represented within a two-dimensional space, enhancing clustering structure explanation based on genetic similarities. This workflow advances accurate intra-host mutation detection, facilitating a deeper understanding of viral diversity and evolution.

## Introduction

The advancements in high-throughput sequencing technologies have revolutionized the study of viral genomes, particularly evident in the case of SARS-CoV-2 during the COVID-19 pandemic. The ability to track the virus’s mutations and evolution during host infection is critical in understanding the emergence of various variants of concern (VOCs). VOCs are defined by their ability to affect viral transmissibility, immune escape, or disease severity, often through key mutations. While most observed mutations will be neutral, arising from stochastic processes such as random genetic drift and founder effects, some mutations may result from selective pressures within an individual host (intra-host) or during transmission between hosts (inter-host) (1). The combined influence of error-prone replication, host RNA-editing mechanisms (2,3), random genetic events, and natural selection form a dynamic landscape that facilitates the emergence of VOCs. Understanding how these forces interact is essential to predict and track the evolution of viral lineages.

In the current literature, there are several hypotheses to explain the interplay between intra-host and inter-host dynamics in the development of SARS-CoV-2 VOCs (4). These hypotheses include evolution within chronically infected individuals (5–12), spillovers from animal populations (13–17), and emergence in regions with limited genomic surveillance. Understanding these processes is vital to explain the rapid evolution of VOCs such as Delta and Omicron, which have shown significant evolutionary leaps.

In response to the pandemic, a vast number of next-generation sequencing (NGS) libraries for SARS-CoV-2 have been generated, primarily to construct consensus sequences for tracking inter-host mutations and VOCs. However, they also provide valuable insights into intra-host diversity, enabling the identification of intra-host single nucleotide variants (iSNVs) that are key in exploring hypotheses around VOC emergence. Despite the considerable size and breadth of available NGS libraries, the existing body of research on iSNV analysis remains limited, with the majority of studies focusing on a relatively small number of NGS libraries (5,6,18–24). This gap in research can also be attributed to challenges related to data quality, such as the presence of sequencing artifacts that introduce errors and lead to false iSNVs.

Preliminary observations have pointed out similar issues, noting, for example, that non-specific amplification in certain sequencing protocols can lead to erroneous mutations that complicate the identification of true intra-host mutations (25). To mitigate these challenges, current practices in intra-host viral analysis include the use of technical replicates (24), which, while effective, are resource-intensive, and the application of hard filters on coverage and frequency, which lack uniformity across different studies and often overlook noteworthy sequencing artifacts like strand bias (21,26–29). This concern underscores the need for more standardized methodologies in processing complex sequencing data to ensure accurate and reliable iSNV analysis.

In computational biology, dimensionality reduction techniques are essential, making the representation of high-dimensional datasets more interpretable and manageable for analysis. This is particularly true for viral sequencing data, which involves thousands of mutations and libraries. These techniques allow to reduce the complexity of the data while preserving meaningful biological signals. Methods like principal component analysis (PCA) (30), commonly used to summarize genetic variation, t-SNE (31,32) for capturing local data structures, and PHATE (33), which excels at visualizing both global and local relationships, are valuable when working with high-dimensional viral genomes. Applying these methods to SARS-CoV-2 data enables the identification of patterns and trends that may be obscured in raw data. While previous studies have primarily applied these techniques to consensus sequences (34–36), they can also be used to analyze the extensive intra-host variation, taking full advantage of their ability to handle high-dimensional genomic data.

Here, we address this gap by using a comprehensive set of publicly available SARS-CoV-2 NGS libraries from the NCBI database, representing the pandemic’s initial years. We use a combination of bioinformatics tools, stringent quality control measures, and dimensionality reduction methods such as PHATE and t-SNE to identify intra-host mutations from sequencing artifacts. Our approach provides a workflow for analyzing SARS-CoV-2 sequencing data and establishes adapted thresholds for the 2020 and 2021 datasets. In this study, we establish a framework for rapid and precise analysis of intra-host viral data, aiming to support pandemic preparedness and response.

## Materials and methods

### Data selection and library pre-processing

We downloaded a set of SARS-CoV-2 Illumina amplicon paired-end sequencing libraries dataset from the first 2 years of the COVID-19 pandemic, ensuring a representative sampling across time and geographic locations. For each month from January 2020 to December 2021, sequencing libraries were randomly chosen based on availability in the National Center for Biotechnology Information (NCBI): up to 5000 from the UK, up to 1000 from the USA, and up to 2000 from other global regions, totalling a potential 8000 libraries monthly (Figure 2A). This yielded a total of 147 537 downloaded libraries (supplementary information section 11.1 and Supplementary Table S2). For each library, Illumina sequencing adapters and bad-quality reads (Phred score < 20) were trimmed from the sequencing reads using TrimGalore V.0.6.0 (https://github.com/FelixKrueger/TrimGalore) (37). The trimmed libraries were mapped to the SARS-CoV-2 reference genome (NC045512.2) using BWA mem v.0.7.17-r1188 (38), generating BAM files. Next, we used the iVar pipeline for primer trimming (39), using a combination of the ARTIC Network V3, V4, and V4.1 amplicon designs, as the sequencing centers in our dataset predominantly use these three kits during the sampling period (https://github.com/artic-network/primer-schemes). We used Samtools mpileup (with specific parameters -Q 20 -q 0 -B -A -d 600000) (40) to generate pileup files containing read information for each BAM file. To parse the pileup files and extract relevant data, we employed the publicly available script pileup2base (https://github.com/riverlee/pileup2base). We calculated the depth of coverage for each genomic position, which is the number of reads aligning to the position. The mean coverage across all libraries is 10446X, so we labelled any position with depth below 100× (1% of the mean) as low-quality. We calculated two metrics to evaluate each library’s quality (Figure 2B): (i) *C*, the mean coverage (the mean number of reads per position) and (ii) *B*, the breadth of coverage (the number of genomic positions with a depth above 100X). We kept the libraries with *C* > 100× and *B* > 20 000 positions (representing two-thirds of the SARS-CoV-2 genome), yielding a total of 128 423 high-quality libraries. (see supplementary information section 11.1 for more details)

**Figure 1.**
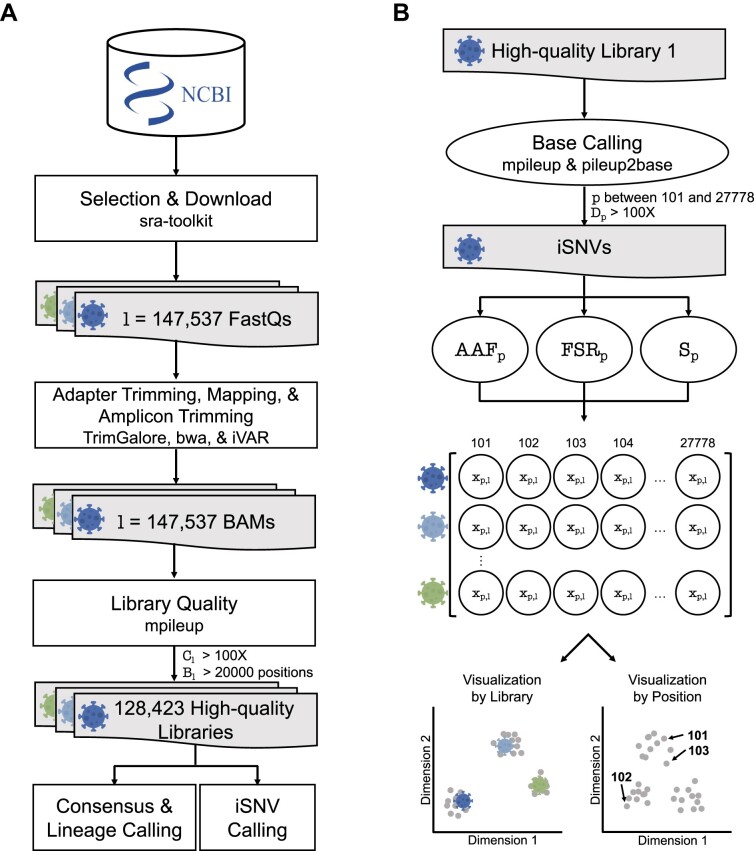
SARS-CoV-2 Sequencing Library Processing Workflows. (**A**) Processing workflow of a set of SARS-CoV-2 sequencing libraries. The workflow starts with selecting and downloading 147 537 FASTQ libraries from NCBI. Next, these libraries were trimmed for adapters, mapped to a reference, and trimmed again for primer targets. We set whole genome coverage filters of mean depth (*C*) >100× and breadth of coverage (*B*) >20 000 on each library *l* to keep only high-quality libraries, keeping 128 423 libraries for further analysis. (**B**) Processing workflow of a single RNA sequencing library. Within each high-quality sequencing library, base calling was done to extract iSNVs. During this process, the ends of the genome were removed, keeping genomic positions *p* between 101 and 27 778 and the depth *D* > 100× of *p*. Subsequently, for each iSNV, we computed the following quality metrics: alternative allele frequency (*AAF*), forward strand ratio (*FSR*) and strand bias likelihood (*S*, equation 1). Thresholds for *AAF* and *S* metrics were established using dimensionality reduction visualization methods, reducing the data into two dimensions by either the libraries (left) or the genomic positions (right).

**Figure 2.**
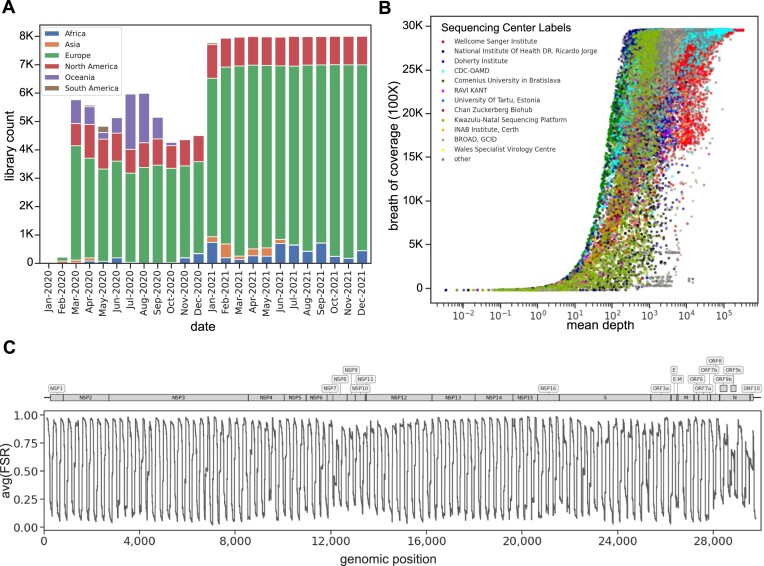
Data description and whole genome quality control. (**A**) The 147 537 Illumina paired-end amplicon sequencing libraries were selected and downloaded from NCBI. Each bar in the graph represents the number of samples, categorized by their respective collection dates, with labels indicating their continents. (**B**) The *x*-axis shows each library’s mean depth of coverage (log scale), while the *y*-axis shows the breadth of coverage. This breadth of coverage is the count of genomic positions covered by at least 100× of depth (*B*). Sequencing centers with at least 1000 libraries (93%) in our dataset are explicitly used as labels, while the remaining libraries have been grouped under the ‘Others’ label. (**C**) Forward strand ratio (*FSR*) averaged across libraries for each genomic position. The gene annotation is overlaid on the top panel.

### Consensus sequences and lineage annotations

We obtained a consensus sequence for each of the 128 423 high-quality libraries using the iVar pipeline consensus calling tool (-q 20 -t 0.75 -m 20) (39). These consensus sequences were annotated with Pango lineages using Pangolin 4.3 (41), which were next used to annotate with World Health Organization (WHO) lineages (Alpha, Delta, Omicron, Delta, Gamma and Others) using a custom script. Sequences with no Pango lineage were annotated as ‘Unassigned’.

### iSNV calling and encoding

We called iSNVs present in the 128 423 high-quality sequencing libraries (Figure 1B) after extracting genomic positions between positions 101 and 29 778, to exclude positions located at both ends of the genome that are generally of lower quality. For each library, we used pileup2base (40) to obtain a base file, which contains, for the 29 678 positions, the counts for each nucleotide (A, T, C, G) separated according to amplicon direction (forward or reverse strand). Because we are focusing our analyses on single nucleotide substitutions, we ignored the number of reads with insertions and deletions. During this step, we kept only positions with a minimum coverage read depth of 100×.

We computed different iSNV metrics at the position level for each library using custom scripts. We define the alternative allele (*AA*) as the most frequent allele at a given position other than the reference allele. For each position and each library, we computed the Alternative Allele Frequency (*AAF*) as *AAF* = (*D*_*AA*_)/*D*, where *D* is the depth at the position studied and *D*_*AA*_ is the depth for the alternative allele.

Due to the nature of targeted sequencing with amplicon design (42), it is possible that a single position in the genome may not be sequenced in a balanced manner between the forward and reverse directions. We thus compute the forward strand ratio as *FSR* = *D*^*f*^/*D*, with *D*^*f*^ the forward strand depth and *D* the total depth.

To evaluate if an alternative allele exhibits unbiased sampling across both strand directions, we used a binomial test. This test determines the probability of observing an allele predominantly on one strand, indicating a higher artifact likelihood. For the forward strand, let *Y* represent the expected count of reads bearing the alternative allele within the total forward strand reads, *D*^*f*^. Assuming *Y* follows a binomial distribution with a probability of success given by the *AAF*, the probability of observing at least $D^f_{AA}$ forward strand reads with the alternative allele (*AA*) is calculated as:


(1) \begin{equation*} S^f(Y \le D^{f}_{AA}) = \sum _{y=0}^{D^{f}_{AA}} \binom{D^{f}}{y} (AAF)^{y} (1-AAF)^{D^{f} - y} \end{equation*}

This same approach is applied to the reverse strand reads, *D*^*r*^, to calculate the likelihood of observing at least $D^r_{AA}$ reverse strand reads with the alternative allele. Finally, to ensure a stringent evaluation, we take the minimum value of these calculated probabilities for both the forward and reverse strands. This minimum value serves as the strand bias likelihood (*S*) metric for each iSNV, effectively quantifying the likelihood of no strand bias, and thus, a low value reflects the potential for the presence of an artifact.

The resulting iSNVs for each sequencing library are represented by their *AAF* given position *p* in a library *l* (*x*_*p*, *l*_) (Figure 1B). This forms a matrix *X*, where the rows are our 128 423 high-quality sequencing libraries, and the columns are the genomic positions between 101 and 27 778. We encode the initial unfiltered matrix *X* with *x*_*p*, *l*_ = *AAF*_*p*, *l*_ for all iSNVs from a given library *l*, and when an iSNV is filtered out based on thresholds for *AAF* and *S*, *x*_*p*, *l*_ is set to 0.

### Dimensionality reduction and clustering evaluation

Given the high dimensionality of matrix *X*, we used dimensionality reduction methods to explore the underlying structure within the high-quality libraries in two dimensions (2D). These techniques allow us to reduce the complexity of the high-dimensional data while preserving essential relationships and patterns, facilitating the identification of meaningful variations across sequencing libraries. They enable us to uncover structure and clustering patterns in the data, which can then be compared to metadata about the sequences, or mutations, to identify potential correlations or underlying trends. We used incremental principal component analyses (PCA) for initialization and then obtained 2D representations of the PCA-transformed data (20 first PCs) using two different approaches: the widely-used t-distributed Stochastic Neighbor Embedding (t-SNE) (32,43,44) and the more recent heat diffusion for affinity-based transition embedding (PHATE) (33). We applied t-SNE with the Python library sklearn.manifold.T-SNE and PHATE with the PHATE Python library (33). The 2D embedding outputs from PHATE and t-SNE are visualized in scatter plots where each library is coloured either by sequencing center or WHO lineage annotation.

To measure the impact of specific subgroups of iSNVs on clustering structures based on either sequencing center or WHO lineage labels, we used a *k*-nearest neighbour (kNN) approach, using *k* = 100. This value of *k* is selected to simplify interpretation as a percentage during neighbour selection and reflects the large number of libraries in our dataset. For each library *l* within a representation, we identify the 100 nearest libraries *NN*(*l*) using the sklearn.neighbors Python package (44). We then calculate *NN*_$z$_(*l*), the count of nearest neighbours sharing the same *z* label as library *l* (where *z* is either *WHO* for lineage or *SC* for sequencing center), and compute the percentage of nearest neighbours with matching labels. For each representation, we derive a final *PNN*_$z$_ as the mean percentage of nearest neighbours with matching labels across all libraries. A higher *PNN*_*z*_ indicates that label *z* describes the data’s clustering structure. We also generate a baseline *PNN*_*z*_, representing expected chance levels by randomly shuffling labels *z* before calculation. This baseline acts as a standard for assessing the significance of observed patterns, emphasizing the delta between observed and baseline *PNN*_*z*_ over the choice of *k* value.

### Experimental design to mitigate sampling biases

We designed a controlled sub-sampling experiment by randomly sub-sampling libraries for each WHO or sequencing center annotation to address the impact of biases stemming from unbalanced sampling. To evaluate the influence of iSNVs on WHO patterns, we iteratively sampled 1000 library rows for each of the Alpha, Beta, Delta and Omicron variants from the data matrix *X* ten times, generating replicates. This process resulted in ten matrices, each comprising 4000 rows. To investigate the effect of iSNVs on sequencing center patterns, we used a similar approach, randomly selecting 1000 library rows. However, in this case, we randomly sampled 1000 library rows from our dataset’s top 10 most frequent sequencing centers (Supplementary Table S3). This process resulted in ten matrices as replicates, each comprising 10 000 rows. Within each matrix, *x*_*p*, *l*_ values were set to 0 based on various *AAF* and *S* thresholds cutoffs. After these two steps, we applied the same method as in Data Visualization to generate PHATE and t-SNE visualization of the matrices. Subsequently, we quantified the clustering structure of t-SNE and PHATE to derive a *PNN*_$z$_ value for each visualization. Specifically, a high value of *PNN*_*SC*_, indicating clustering primarily by sequencing center, would suggest a dataset enriched for artifacts. Conversely, a high value of *PNN*_*WHO*_, signifying clustering primarily by WHO lineage annotations, would suggest a more biologically relevant dataset.

### Mutational load

The mutational load for each library was calculated as the total count of distinct iSNVs identified regardless of allele frequencies. Per library, mutational loads were visualized using histograms to illustrate the distribution of mutational loads across the dataset. We categorized the libraries into different percentiles based on their mutational load, identifying those with higher or lower numbers of iSNVs.

### Substitution spectrum analysis

To assess the mutational landscape and identify specific patterns that may indicate underlying mutational mechanisms or biases in the dataset, we looked at the substitution patterns within iSNVs’ different *AAF* frequencies. First, we categorized intra-host iSNVs into four *AAF* bins, as follows: 5–25%, 25–50%, 50–75% and 75–100%. This categorization was based on the evidence for an alternative allele present in the iSNVs. Next, within each *AAF* bin, we classified each iSNV in terms of its ancestral allele and alternative allele to obtain 12 categories of substitution types. These are A>G, A>C, A>T, C>A, C>G, C>T, G>A, G>C, G>T, T>A, T>C and T>G. This allowed us to analyze the relative contribution of each substitution type within each *AAF* range.

## Results

### Curation pipeline overview

While NGS data offers valuable insights into viral diversity and evolution, extracting meaningful information demands rigorous bioinformatics and representation approaches. A systematic methodology is crucial to process this data accurately, ensuring the reliability of identified iSNVs. We, therefore, propose a comprehensive workflow to extract meaningful intra-host mutations from NGS data. Our workflow is divided into two levels (Figure 1): the processing and quality control of a set of libraries (Figure 1A) and the processing and quality control of iSNVs within each library (Figure 1B).

To build a set of high-quality libraries, we meticulously processed a large set of Illumina amplicon paired-end sequencing libraries, ensuring a representative sample across various time points and locations. The data processing includes adapter and quality trimming, alignment to the SARS-CoV-2 reference genome, primer trimming, and whole genome coverage quality control (see Method section 2.1). Using the processed libraries, we performed iSNV calling and computed key metrics such as Alternative Allele Frequency (*AAF*) and Strand bias likelihood (*S*) (see Materials and methods). These metrics help to accurately identify putative iSNVs while minimizing technical artifacts. Next, dimensionality reduction methods, such as PHATE and t-SNE, are applied to visualize and interpret the iSNV data through analyses of clustering structures. In this process, we generate representations in two distinct ways: by the library, where each point in the visualization represents a summary of the library, and by genomic position, where each point corresponds to a specific genomic position summarizing its behaviour across libraries. PHATE maintains meaningful distance between clusters (33), preserving hierarchical relationships between sequencing libraries. We, therefore, use this PHATE representation to present our main results. Similar findings are observed using t-SNE (see supplementary information section 11.5). To differentiate between potential artifacts and biologically relevant patterns, the clustering structures are measured using the percentage of nearest neighbors (*PNN*) presenting the same lineage label (as defined by the World Health Organization, WHO) or sequencing center (SC), providing a robust metric to quantify clustering structures of different sets of iSNVs. While the null hypothesis is for iSNVs to be randomly distributed, SC labels are used to check for associations with sequencing centers as a proxy to identify potential artifacts. Conversely, WHO labels are expected to reflect biological relevance, with the limitation that some sequencing centers favour the sequencing of some lineages over others. The variation in lineage sequencing by some centers is influenced by the differing prevalence of these lineages across regions.

### Extracting emerging *de novo* iSNVs

We processed 128 423 high-quality SARS-CoV-2 sequencing libraries from the first two years of the COVID-19 pandemic, ensuring a representative sampling across time and geographic locations (Figure 2A). Genome data quality was assessed by coverage depth (Figure 2B, *x*-axis), breadth of coverage (Figure 2B, *y*-axis), and strand balance (Figure 2C). The distribution of the depth and breadth of coverage reveals center-specific quality variability. In turn, the strand balance shows unbalanced strand coverage across the genome with an oscillating pattern at the same genomic regions independent of the sequencing center (see supplementary information section 11.2).

**Figure 3.**
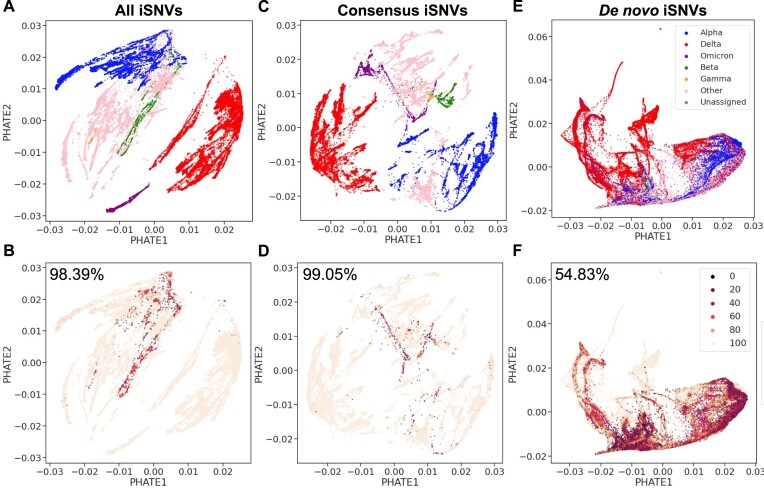
The PHATE representation organizes the unfiltered SARS-CoV-2 libraries according to WHO lineage annotation. (A, B) Represents the full iSNV dataset; (C, D) includes only consensus iSNVs; (E, F) contains only *de novo* iSNVs. (**A**) PHATE visualization of the full dataset matrix with 11 635 668 iSNVs, using WHO lineage labels. (**B**) The same dataset as (A), labelled with the percentage of nearest neighbours (*k* = 100) that share the same WHO annotation as the library itself, and the total *PNN*_*WHO*_ value is displayed at the top left. Darker-coloured points signify a lower percentage of neighbouring points sharing the same label as the focal point. (**C**) PHATE visualization of the consensus matrix, containing 3 634 563 iSNVs, with WHO lineage labels. (**D**) Consensus matrix, similar to (C), but with *PNN*_*WHO*_ labelling and the *PNN*_*WHO*_ value at the top left. (**E**) PHATE visualization of the *de novo* matrix, including 8 000 668 iSNVs, labelled with WHO lineage annotations. (**F**) *de novo* iSNV matrix, as in (E), but with *PNN*_*WHO*_ labelling and the *PNN*_*WHO*_ value at the top left. Where a lineage lacks a WHO designation, it is labelled as ‘Other’ and unassigned lineages are labelled as ‘Unassigned.’

We identified a total of 11 635 668 iSNVs in these libraries before any filtering steps, averaging 91 iSNVs per library (see Methods section 2.3 and Supplementary Table S1). PHATE representation of the raw iSNV dataset distinctly discriminates libraries according to WHO lineage annotations (Figure 3A). The mean Percentage of Nearest Neighbours from the same WHO lineage (*PNN*_*WHO*_) (Figure 3B) is at 98.39%, corroborating a strong lineage-specific signature in the raw iSNVs (see Methods section 2.4).

This result is likely driven by lineage-specific mutations, herein referred to as consensus iSNVs, which are identified as having an *AAF* over 75% and are usually part of consensus sequences (45–47). These consensus iSNVs account for 3 634 563 iSNVs, averaging 28 per library (Supplementary Table S1), aligning with the SARS-CoV-2 mutation rate reported by NextStrain (48) for this time period highlighting the reliability of these consensus iSNVs. PHATE representation of consensus iSNVs only again shows strong alignment with WHO lineages (Figure 3C), which is reflected in the high *PNN*_*WHO*_ values in PHATE of 99.05% (Figure 3D), confirming these iSNVs largely drive the lineage-specific clustering observed in our raw iSNV dataset.

In contrast, we define putative *de novo* iSNVs (*AAF* < 0.75), representing emerging viral mutations within the host, totalling 8 000 668 in the raw dataset, averaging 62 per library (Supplementary Table S1). The *de novo* iSNVs exhibit more heterogeneous clustering patterns (Figure 3E) with a lower *PNN*_*WHO*_ value of 54.83% (Figure 3F), suggesting a less pronounced lineage-based structure. However, the clustering patterns of the *de novo* iSNVs show a stronger alignment with lineage structure than expected by chance, with the baseline *PNN*_*WHO*_ from random resampling at 32.82% for PHATE representation. This highlights the significance of the observed *PNN*_*WHO*_ compared to the baseline value, suggesting a lineage-specific biological relevance in the emerging mutations. The controlled sub-sampling experiments (Supplementary Figure S1, detailed in Methods section 2.5 and in supplementary information section 11.4) further support these observations, underscoring the distinct clustering behaviours of consensus and *de novo* iSNVs.

### Resolving artifacts in *de novo* iSNVs

**Figure 4.**
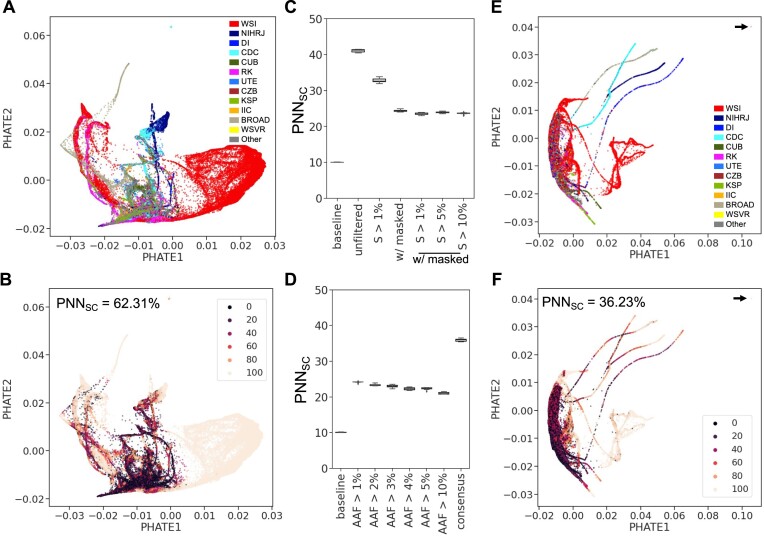
The use of *S* and *AAF* metrics improves SARS-CoV-2 *de novo* iSNVs’ PHATE structure by mitigating sequencing center batch effects and artifacts. (**A**) PHATE visualization of the unfiltered *de novo* matrix containing 8 000 668 iSNVs, labelled by the libraries’ sequencing centers. (**B**) PHATE visualization as in (A), but with labels showing the percentage of *k* = 100 nearest neighbours that share the same sequencing center annotation as the library itself and the total *PNN*_*SC*_ value is displayed at the top left. (C and D) Boxplots displaying *PNN*_*SC*_ values for each PHATE visualization, derived from a sub-sampling controlled experiment across ten replicates (see Method section 2.5). (**C**) Shows *PNN*_*SC*_ values across various *S* metric thresholds, and (**D**) presents *PNN*_*SC*_ values across different *AAF* metric thresholds. (**E**) PHATE visualization of the *de novo* matrix, filtered based on *S* and *AAF* thresholds, labelled by sequencing centers. The arrow points to a set of libraries that seem to diverge from the main cluster. (**F**) PHATE visualization as in (**E**), but with labels showing *PNN*_*SC*_ values, and the total *PNN*_*SC*_ value is displayed at the top left. In this representation sequencing centers with at least 1000 libraries in our dataset are explicitly labelled with its sequencing center as follows: Welcome Sanger Institute (WSI), National Institute of Health DR. Ricardo Jorge (NIHRJ), Doherty Institute (DI), CDC-OAMD (CDC), Comenius University in Bratislava (CUB), Ravi Kant (RK), University of Tartu in Estonia (UTE), Chan Zuckerberg Biohub (CZB), Kwazulu-Natal Sequencing Platform (KSP), INAB Insitute in Certh (IIC), BROAD GCID (BROAD), Wales Specialist Virology Center (WSVR). While the remaining libraries were grouped under the ‘Other’ label.

Due to the geographic distribution of lineages, sequencing centers often sequence certain lineages more frequently than others, potentially leading to technical artifacts that affect lineage clustering in the *de novo* iSNV subset. This is confirmed by the clustering analysis of libraries containing only the 8 000 668 *de novo* iSNVs (Figure 4), where the PHATE representation showed significant sequencing center batch effects (Figure 4A), with a mean percentage of nearest neighbours from the same sequencing center (*PNN*_*SC*_) value of 62.31% (Figure 4B), greatly exceeding the baseline value of 27.53%, expected by chance. This result indicates that our set of *de novo* iSNVs likely contains sequencing artifacts. To filter out sequencing artifacts from the set of *de novo* iSNVs and refine the dataset, we used the strand bias metric *S* (see Materials and methods, equation 1) and the *AAF* metric.

The PHATE visualization of the unfiltered *de novo* iSNVs prominently identifies the Wellcome Sanger Institute as a major cluster (Figure 4A) due to its significant representation of 75% in our library set. This underscores the potential impact of unbalanced sampling on cluster formation and potentially *PNN*_*SC*_ values. To neutralize this imbalance, we designed a controlled sub-sampling experiment, evenly selecting 10 000 libraries from each of the top 10 sequencing centers based on library counts (see Method section 2.5), aiming to reduce the impact of sampling bias on the *PNN*_*SC*_ values. We thus assessed the impact of filtering based on these two metrics, *S* and *AAF*, on the PHATE clustering structure measured with *PNN*_*SC*_ using the controlled sub-sampling experiment to mitigate bias from uneven sampling across sequencing centers (Figure 4C, D).

To address the observed strand coverage imbalance in our dataset (Figure 2C), we used the strand bias metric (*S*), which assesses the likelihood of strand bias artifacts using the alternative allele’s strand coverage. Initially, filtering out iSNVs with *S* < 1% and 486 genomic positions showing recurrent strand bias across libraries (see supplementary information section 11.3) significantly lowers sequencing center-specific artifacts. This was reflected in the reduced *PNN*_*SC*_ values (Figure 4C) in the controlled sub-sampling experiments. However, *PNN*_*SC*_ values remained stable when the *S* threshold was increased beyond 1%, suggesting no further improvement based on this metric (Figure 4C).

Filtering based on allele frequency is a key metric in genomic studies. Some studies use a low threshold (21,23,29,49,50), which may result in the inclusion of erroneous intra-host mutations. In contrast, more stringent criteria could overlook the analysis of low-frequency, *de novo* intra-host mutations. Further refinement of our iSNV set based on the *AAF* metric led to an additional decrease in *PNN*_*SC*_ values (Figure 4D), particularly when increasing the *AAF* threshold to 5%. Despite testing additional combinations of thresholds, the final *PNN*_*SC*_ metric did not reach the baseline value of 10%, suggesting that the optimal threshold on the *AAF* metric is 5%.

Applying these optimal thresholds of 1% for *S* and 5% for *AAF* to filter out iSNVs, the *de novo* iSNV count dropped from 8,000,668 to 468,651, averaging six iSNVs per library (Supplementary Table S1). This process notably decreased sequencing center batch effects in the PHATE representation (Figure 4E, F), resulting in a *PNN*_*SC*_ value of 36.23%. While this value still exceeds the baseline of 27.69%, the reduction marks an improvement in minimizing batch effects. Additionally, the *PNN*_*SC*_ value for lineage-defining consensus iSNVs also does not reach the baseline value (Figure 4D), implying that completely separating sequencing center influences from lineage-specific signatures might represent an intractable challenge.

### Identifying outliers and center-specific patterns

In our analysis of the 468 651 filtered *de novo* iSNVs, there remain outlier clusters showing sequencing center homogeneity in the PHATE representation (Figure 4E, F). Notably, a small but distinct set of libraries forms an outlier cluster, markedly separated from other libraries in the PHATE representation (Figure 4E, F, indicated by an arrow). This observation suggests that specific libraries from the same sequencing centers potentially have an excess of shared iSNVs. We thus analyzed libraries’ intra-host mutational load, defined as the number of iSNVs in a library (see Method section 2.6). While most libraries in our dataset contain only one or two iSNVs (Supplementary Figure S2), some exhibit a high intra-host mutational load, with tens of iSNVs per library.

To determine the optimal threshold for excess iSNVs in libraries, we computed the *PNN*_*SC*_ value in PHATE representation after sequentially removing the top 1%, 5% and 25% of the most mutated libraries (Supplementary Figure S2A, B). Removing the top 1% of outliers impacted the *PPN*_*SC*_ value the most, decreasing it by 2%. Additional exclusions, even down to only keeping libraries with one iSNV, did not further reduce the *PNN*_*SC*_ value (Supplementary Figure S2B, 50th percentile), underlining the impact of extreme outliers in the PHATE representation of the full dataset.

**Figure 5.**
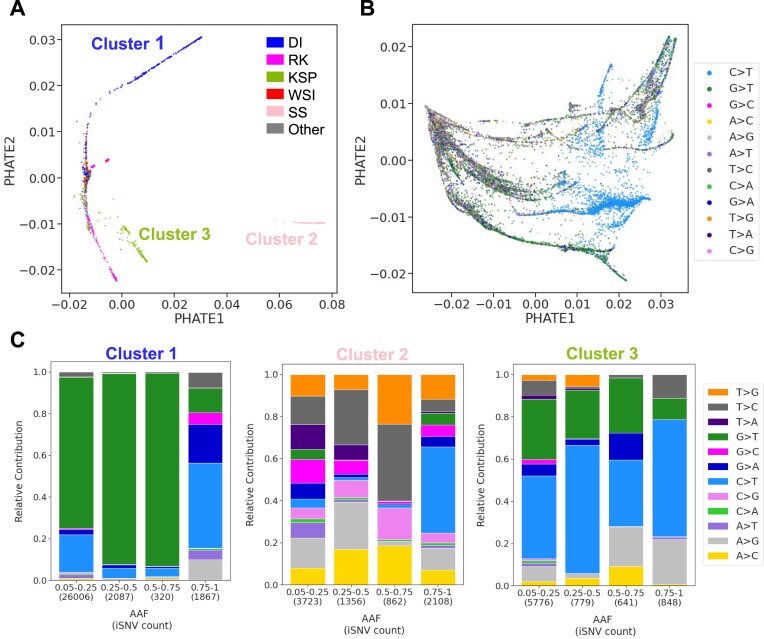
Unique mutational patterns in SARS-CoV-2 outlier libraries tied to sequencing centers. (**A**) PHATE visualization of outlier libraries, showcasing distinct clusters of SARS-CoV-2 libraries, each associated with specific sequencing centers. (**B**) Displays the PHATE representation of genomic positions in outlier libraries, labelled with the most frequent substitution types observed across these libraries. (**C**) Mutational patterns in iSNVs across the three distinct clusters in (A), each associated with a specific sequencing center. Each mutational pattern figure sequentially presents mutational patterns in iSNVs from Cluster 1, sequenced by the Doherty Institute; Cluster 2, associated with Scilifelab Stockholm; and Cluster 3, predominantly sequenced by the Kwazulu-Natal Sequencing Platform. The sequencing center’s labels are as follows: Welcome Sanger Institute (WSI), Doherty Institute (DI), Ravi Kant (RK), Kwazulu-Natal Sequencing Platform (KSP) and Scilifelab Stockholm (SS).

To ensure biologically relevant libraries are not excluded, we explored in-depth the patterns observed in the top 1% outlier libraries (1,270 outlier libraries) by computing the PHATE representation only on these libraries (Figures 5A, Supplementary Figure S3A). Those strongly clustered by sequencing centers indicate that their iSNVs are enriched for sequencing center-specific artifacts. In this PHATE representation of the outlier libraries, we note four main clusters (Supplementary Figure S3). Cluster 1 is composed of 159 Doherty Institute libraries, corresponding to Australia’s first pandemic wave (March–August 2020) (Supplementary Figure S3B). Cluster 2 comprises 104 libraries from Scilifelab Stockholm, collected at the end of the second pandemic wave (December 2020–February 2021). Cluster 3 includes 109 libraries from the Kwazulu-Natal Sequencing Platform, with collections from January to April 2021. Cluster 4 comprises 75 libraries from the Ravi Kant sequencing center, with a collection peak in May 2021.

**Figure 6.**
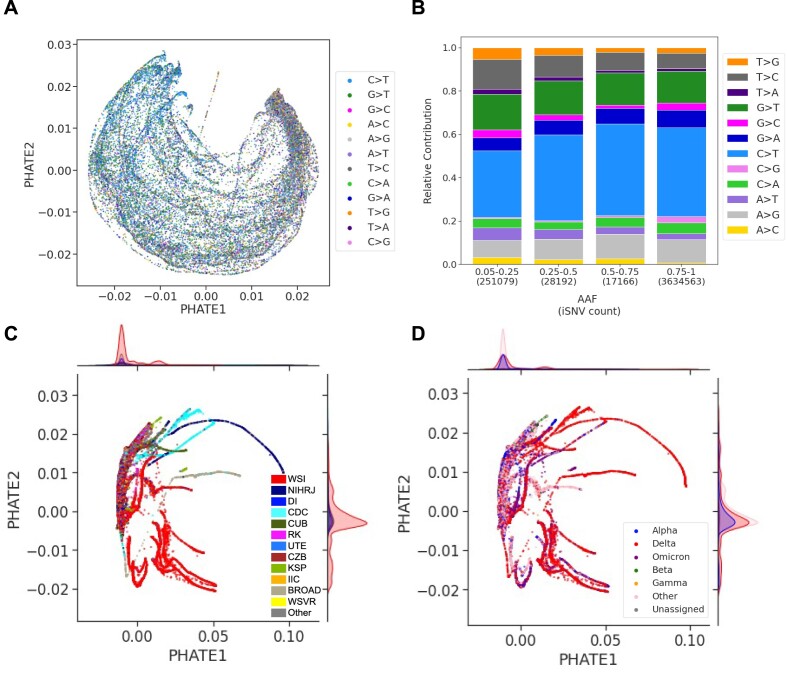
Attaining a refined and comprehensive collection of SARS-CoV-2 intra-host sequencing libraries and iSNVs via meticulous filtering. (**A**) PHATE visualization of the refined library set, excluding outliers, with *de novo* iSNVs filtered based on *S* and *AAF* metrics. Each library is labelled by its sequencing center. (**B**) Similar to (A), but with labels showing the percentage of nearest neighbours (*PNN*_*SC*_) for each sequencing center and the total *PNN*_*SC*_ value displayed at the top left. (**C**) The total 296 437 *de novo* and consensus iSNVs, stratified by *AAF* and substitution types to reveal mutational biases. (**D**) Presents a PHATE visualization of the transposed matrix for non-outlier libraries with filtered *de novo* iSNVs. Each point represents a genomic position of the SARS-CoV-2 genome, labelled by its most frequent substitution type across the libraries. Sequencing centers with at least 1000 libraries are explicitly labelled with its sequencing center as follows: Welcome Sanger Institute (WSI), National Institute of Health DR. Ricardo Jorge (NIHRJ), Doherty Institute (DI), CDC-OAMD (CDC), Comenius University in Bratislava (CUB), Ravi Kant (RK), University of Tartu in Estonia (UTE), Chan Zuckerberg Biohub (CZB), Kwazulu-Natal Sequencing Platform (KSP), INAB Insitute in Certh (IIC), BROAD GCID (BROAD), Wales Specialist Virology Center (WSVR).

To detect the mutational patterns responsible for these effects, we computed PHATE representation by genomic position on these outliers libraries (Figure 5B), showing clustering of C>T and G>T mutations. This contrasts with non-outlier libraries, which do not show clear clustering based on substitution patterns (Figure 6A). Quantifying the proportion of iSNVs based on the substitution spectrum (see Method section 2.7) revealed unique mutational signatures within each of the three main clusters with the most libraries (Figure 5C). Each cluster, associated with a specific sequencing center, exhibited mutational patterns distinct from those in non-outlier libraries (Figure 6A). Cluster 1 displays a prominent G>T pattern in *de novo* iSNVs, not seen in the consensus iSNVs from these same sequences. Interestingly, we identified 40 genomic positions with a *de novo* iSNV in at least 80% of the libraries in cluster 1. Cluster 2 libraries also displayed a unique mutational pattern in their iSNVs (Figure 5C, center), with T>G, T>C, A>G and A>C as the predominant substitutions. These also diverged from their respective consensus iSNVs except for T>G. A notable 30 genomic positions have a *de novo* iSNV in at least 80% of the libraries in cluster 2. Lastly, cluster 3 libraries presented an excess of G>T and C>T that differed from their consensus iSNVs. In this cluster 3, 14 genomic positions have a *de novo* iSNV in at least 80% of the libraries.

Overall, our outlier analysis revealed unique mutational patterns in *de novo* iSNVs across different sequencing centers associated with an excess of iSNVs, showing the influence of center-specific sequencing factors. These findings confirm the need to filter out the top 1% outlier libraries with a mutational load above 44 iSNVs in our library set. Our results also highlight the importance for sequencing centers to assess both the abundance of iSNVs and the presence of unique mutational patterns as key indicators for evaluating their sequencing processes.

### Deriving a final *de novo* iSNV dataset

After our extensive curation, we kept 296 437 *de novo* iSNVs with $AAF>5\%$ and $S>1\%$, from 72 470 non-outlier libraries with at least one iSNV, as our final curated dataset. The PHATE visualization of the 296 437 retained *de novo* iSNVs by genomic position display no clustering according to the mutational pattern, underscoring the optimal curation of the dataset (Figure 6A). Additionally, the substitution spectrum of this curated set shows a prevalence of C>T and G>T substitutions (Figure 6B), aligning with consensus iSNV patterns and known SARS-CoV-2 mutational trends (51–54).

The PHATE visualization by library (Figure 6C) shows greater sequencing center homogeneity compared to the initial representation of the raw iSNV data (Figure 4A). WHO lineage annotation of the same PHATE representation shows similar lineage homogeneity (Figure 6D). Both sequencing center and WHO lineage annotations in the PHATE representation concentrate the majority of libraries into a single large cluster, as shown by the density plots. Despite the presence of sequencing center-specific clusters (Figure 6C), lineage-specific clustering is also noticeable (Figure 6D), suggesting that lineages from similar geographic regions may share iSNV generation processes, meriting further investigation. Nevertheless, the optimal refinement of the dataset is supported by a substantial decrease in the *PNN*_*SC*_ value, from 62.31% to 33.26% (baseline value 26.29%).

## Discussion

Emerging *de novo* mutations, or iSNVs, which occur during the intra-host phase of infection, are critical for understanding viral diversity and evolution. These mutations can be detected by analyzing sequencing libraries from infected hosts, although the sequencing process may introduce artifacts, resulting in false iSNV calls. To address this challenge, we present a comprehensive two-step workflow tailored for intra-host viral NGS analysis, specifically focusing on the SARS-CoV-2 RNA-seq libraries. It is specifically designed to robustly accommodate and correct for artifacts arising from the diverse sources present in our heterogeneous dataset, ensuring accurate detection of true iSNVs. First, we processed a large dataset of libraries with stringent whole genome quality control. Subsequently, we use these libraries for iSNV calling, employing specific quality metrics to differentiate putative iSNVs from artifacts. We also implemented dimensionality reduction techniques like PHATE and t-SNE to visualize and analyze library structures, enhancing our analysis with an explainability metric. Applying this workflow to a substantial SARS-CoV-2 dataset, we identified a set of emerging (*de novo*) iSNVs for studying intra-host viral evolution, differentiating them from consensus iSNVs using a 75% allele frequency threshold. This threshold is often used for its balance between detecting true positives and minimizing false positives, at the expense of intra-host diversity, by consensus callers (45–47). Additionally, we tackled the challenge of distinguishing *de novo* iSNVs from similar-frequency artifacts using tailored quality metrics to establish appropriate thresholds for a given dataset, ensuring our process is rigorous and non-arbitrary.

Sequencing accuracy is influenced by multiple factors, including sample preparation, PCR amplification, and sequencing errors (39,55–57). This is especially the case when accurately detecting viral intra-host diversity (56–58). Mutations appearing on only one strand are likely due to amplification errors, as putative mutations would be present on both strands. Known as strand bias artifacts, they have been overlooked in the literature (56,58,59), but when addressed in recent studies, it is typically through applying a stringent filter that counts the appearances of an alternative allele on each strand (18,20,60). However, this common filtering approach fails to account for the inherent imbalances in strand coverage frequently observed in targeted sequencing of SARS-CoV-2. This oversight can significantly increase the risk of false negatives, with the rate of missed variants varying unevenly across the genome. In response, our strand bias metric takes a different approach by assessing the distribution of each iSNV’s alternative allele across both strands, explicitly accounting for the imbalance in strand coverage observed in our SARS-CoV-2 NGS libraries. This approach avoids the bias of traditional methods that only retain genomic positions covered by both strands, a restriction that could impact about two-thirds of the genome (Figure 2C). Additionally, our strand bias metric, while similar to a published formula (61), is tailored to a large viral NGS dataset. Interestingly, we highlight a set of genomic positions frequently identified as strand bias artifacts supported by our large and comprehensive dataset and see supplementary information section 11.3). By masking these positions, we noted a significant reduction in sequencing center batch effects, indicating that these positions may be specific to sequencing centers. Therefore, we highly recommend masking these positions to mitigate sequencing errors and erroneous intra-host data analysis and provide an efficient way to do so (62).

As intra-host viral genomic data grows in size and complexity (63,64), the challenge of managing these datasets increases. Dimensionality reduction methods are valuable for distilling this data into a more manageable form (65,66). However, interpreting these methods’ two-dimensional representations can be challenging due to unclear biological significance (67). In our workflow, we have incorporated PHATE and t-SNE alongside a metric that computes the percentage of nearest neighbours sharing the same annotation (e.g. sequencing center, WHO variant). This approach enhances the explainability of these techniques by highlighting relationships within specific groups of libraries in the representation, establishing a novel approach to analyzing high-dimensional viral sequence data. This methodology also facilitates the identification of optimal iSNV filtering thresholds, a critical aspect of sequencing data quality control. Implementing this approach allowed us to refine our quality metrics, resolve sequencing center batch effects, and improve the reliability of our iSNV dataset. Moreover, we have pioneered the use of PHATE in viral sequencing data analysis. We show that PHATE is especially effective at handling libraries with varying iSNV counts, unlike t-SNE, which is impacted by such libraries (see Supplementary Figure S2 and supplementary information section 11.5). PHATE’s ability to accurately represent *de novo* mutations also demonstrates its potential for broader applications in areas requiring *de novo* mutation analysis, including the study of cancer clonal mutations (68), evolutionary developmental biology (69), and metagenomics (70).

Despite PHATE’s ability to handle libraries with varying iSNV counts, outlier libraries containing a large number of iSNVs significantly skewed the PHATE clustering structure, highlighting a problematic aspect where a small subset disproportionately impacts the overall analysis. The significant influence of these outlier libraries was apparent in the unique C>T and G>T mutational patterns observed in PHATE’s genomic position representation within the outlier only libraries (Figure 5B), supporting the need to treat these libraries separately. Additionally, the strong clustering by sequencing center of the top 1% outlier libraries suggests that iSNVs within these libraries include sequencing artifacts specific to each center. This was confirmed by the distinct mutational patterns and the recurrence of genomic positions enriched for iSNVs within each outlier cluster, which, in turn, are associated with different sequencing centers. The unique mutational signatures identified within the outlier clusters also provide insight into the potential mechanisms of error introduction or bias in sequencing workflows. For instance, the G>T substitution pattern seen in the Doherty Institute libraries at the beginning of the pandemic (March–August 2020) may signal RNA degradation. Following the adoption of improved sample storage protocols, this institute noted a reduction in the count of observed mutations (Doherty Institute platform members, personal communication). This change in sequencing libraries’ quality emphasizes the necessity of ongoing collaboration between sequencing centers and data analysts to adapt practices and enhance sequencing data accuracy and reliability in real-time.

Our approach, comprehensive as it is, faces some limitations. First, despite documented instances of mixed infections (71,72) where lineage-defining mutations appear at low frequencies, our current workflow is not designed to effectively capture these variations. In cases of mixed infections, iSNVs characterized as *de novo* under our definition may actually stem from the co-presence of two (or more) different strains within a host, as they would fall below our 75% threshold for emerging mutations. Therefore, particular care should be taken when analyzing datasets where a substantial proportion of samples could be mixed infections. In our analysis, we found little evidence of mixed infections, but it remains a possibility that some libraries—and consequently, iSNVs—could originate from such infections, though they would likely have been excluded during our outlier analysis. Furthermore, our workflow is currently not specifically designed to address the complexity associated with calling insertions and deletions (indels), which is an area for future development. In particular, a benchmark of indel detection tools for intra-host data should be conducted to enhance this aspect of viral genomic analysis. Bridging this gap poses a notable challenge and offers a valuable opportunity for methodological innovation. This workflow is designed specifically for Illumina sequencing data, favoured for its lower error rate (73), and is less suitable for Nanopore sequencing (74,75). The latter’s high error rate of about 10% complicates the detection of low-frequency emerging mutations. Our dataset, while diverse, primarily consists of libraries from European and North American sources, mirroring the availability of publicly accessible sequencing data (63). This situation underscores the need for improved sequence sharing and support for sequencing capabilities in underserved regions. Additionally, our reliance on publicly available single instances of sequencing libraries leverages accessible data but complicates the confirmation of variant calls due to the absence of multiple replicates, as done previously. We address this by setting a minimum allele frequency threshold of 5%, higher than the typical Illumina error rate of 1% (73), aligning with the literature that advocates stricter thresholds for variant identification in the absence of replicates (26,39).

Nonetheless, our workflow and dataset of high-quality intra-host iSNVs have proven instrumental in testing biological hypotheses and drawing conclusions on diverse areas of study. Published applications include uncovering immune evasion mechanisms in SARS-CoV-2 through sequence analysis and epitope mapping (60), comparing intra-host viral evolution between immunosuppressed patients and the general population (75), and investigating intra-host mutations that influence epitope binding predictions (76). Additionally, this workflow and the identified set of *de novo* mutations open up new avenues for exploring hypotheses concerning viral intra-host diversity and evolution, providing a foundation for broader research initiatives in this field. For example, we observed that intra-host library clustering based on WHO variants persisted above baseline levels even after removing lineage-defining mutations. This leads us to hypothesize that lineage-defining genetic factors may contribute to the intra-host mutational patterns, suggesting a complex underlying mechanism of viral evolution within hosts. Our methodology has proven robust in detecting these subtle lineage characteristics despite variations in sample distribution, reinforcing the possibility of variant-specific effects on mutational events, a finding supported by a recent study (77). This intriguing result warrants further investigation that could lead to the discovery of lineage dynamics and mutation impacts.

In conclusion, our robust viral intra-host processing and analysis workflow enhances the use of existing cross-sectional sequencing libraries and improves the accuracy and depth of viral genomic analyses. Future directions can include analyzing sequences from wastewater, an alternative approach to continued surveillance of emerging viral mutations on a broader population level (78). This advanced bioinformatics methodology is crucial for deepening our understanding of intra-host diversity and strengthening preparedness strategies for future pandemics, proving essential for responding effectively to other viruses in forthcoming outbreaks.

## Supplementary Material

lqae145_Supplemental_File

## Data Availability

The processing workflow’s code can be found here: https://doi.org/10.5281/zenodo.13910333. NCBI accession IDs used in this study and the high-quality iSNVs identified within each sequencing library are accessible through a Mendeley data repository ((62), https://doi.org/10.17632/8nvgtrkzdm.1). We also provide the list of recommended 477 genomic positions to mask in the same data repository (see supplementary information section 11.3).

## References

[1] Lauring A.S. Within-host viral diversity: a window into viral evolution. Annu. Rev. Virol. 2020; 7:63–81. 32511081 10.1146/annurev-virology-010320-061642 PMC10150642

[2] Di Giorgio S., Martignano F., Torcia M.G., Mattiuz G., Conticello S.G. Evidence for host-dependent RNA editing in the transcriptome of SARS-CoV-2. Sci. Adv. 2020; 6:eabb5813. 32596474 10.1126/sciadv.abb5813 PMC7299625

[3] Nakata Y., Ode H., Kubota M., Kasahara T., Matsuoka K., Sugimoto A., Imahashi M., Yokomaku Y., Iwatani Y. Cellular APOBEC3A deaminase drives mutations in the SARS-CoV-2 genome. Nucleic Acids Res. 2023; 51:783–795. 36610792 10.1093/nar/gkac1238 PMC9881129

[4] Markov P.V., Ghafari M., Beer M., Lythgoe K., Simmonds P., Stilianakis N.I., Katzourakis A. The evolution of SARS-CoV-2. Nat. Rev. Microbiol. 2023; 21:361–379. 37020110 10.1038/s41579-023-00878-2

[5] Sonnleitner S.T., Prelog M., Sonnleitner S., Hinterbichler E., Halbfurter H., Kopecky D. B.C., Almanzar G., Koblmüller S., Sturmbauer C., Feist L. et al. Cumulative SARS-CoV-2 mutations and corresponding changes in immunity in an immunocompromised patient indicate viral evolution within the host. Nat. Commun. 2022; 13:2560. 35538074 10.1038/s41467-022-30163-4 PMC9090742

[6] Quaranta E.G., Fusaro A., Giussani E., D’Amico V., Varotto M., Pagliari M., Giordani M.T., Zoppelletto M., Merola F., Antico A., Stefanelli P., Terregino C., Monne I. SARS-CoV-2 intra-host evolution during prolonged infection in an immunocompromised patient. Int. J. Infect. Dis. 2022; 122:444–448. 35724829 10.1016/j.ijid.2022.06.023 PMC9212919

[7] Hill V., Du Plessis L., Peacock T.P., Aggarwal D., Colquhoun R., Carabelli A.M., Ellaby N., Gallagher E., Groves N., Jackson B. et al. The origins and molecular evolution of SARS-CoV-2 lineage B.1.1.7 in the UK. Virus Evol. 2022; 8:veac080. 36533153 10.1093/ve/veac080 PMC9752794

[8] Ghafari M., Liu Q., Dhillon A., Katzourakis A., Weissman D.B. Investigating the evolutionary origins of the first three SARS-CoV-2 variants of concern. Front. Virol. 2022; 2:942555.

[9] Oude Munnink B.B., Sikkema R.S., Nieuwenhuijse D.F., Molenaar R.J., Munger E., Molenkamp R., van der Spek A., Tolsma P., Rietveld A., Brouwer M. et al. Transmission of SARS-CoV-2 on mink farms between humans and mink and back to humans. Science. 2021; 371:172–177. 33172935 10.1126/science.abe5901 PMC7857398

[10] Hale V.L., Dennis P.M., McBride D.S., Nolting J.M., Madden C., Huey D., Ehrlich M., Grieser J., Winston J., Lombardi D. et al. SARS-CoV-2 infection in free-ranging white-tailed deer. Nature. 2022; 602:481–486. 34942632 10.1038/s41586-021-04353-x PMC8857059

[11] Oreshkova N., Molenaar R.J., Vreman S., Harders F., Oude Munnink B.B., Hakze-van der Honing R.W., Gerhards N., Tolsma P., Bouwstra R., Sikkema R.S. et al. SARS-CoV-2 infection in farmed minks, the Netherlands, April and May 2020. Euro Surveill. 2020; 25:2001005. 32553059 10.2807/1560-7917.ES.2020.25.23.2001005 PMC7403642

[12] Bashor L., Gagne R.B., Bosco-Lauth A.M., Bowen R.A., Stenglein M., VandeWoude S. SARS-CoV-2 evolution in animals suggests mechanisms for rapid variant selection. Proc. Natl. Acad. Sci. U.S.A. 2021; 118:e2105253118. 34716263 10.1073/pnas.2105253118 PMC8612357

[13] Washburne A., Jones A., Zhang D., Deigin Y., Quay S., Massey S.E. Statistical challenges for inferring multiple SARS-CoV-2 spillovers with early outbreak phylodynamics. 2022; bioRxiv doi:13 October 2022, preprint: not peer reviewed 10.1101/2022.10.10.511625.

[14] Sacchetto L., Chaves B.A., Costa E.R., de Menezes Medeiros A.S., Gordo M., Araújo D.B., Oliveira D. B.L., da Silva A. P.B., Negri A.F., Durigon E.L. et al. Lack of evidence of severe acute respiratory syndrome coronavirus 2 (SARS-CoV-2) spillover in free-living neotropical non-human primates, Brazil. Viruses. 2021; 13:1933. 34696363 10.3390/v13101933 PMC8540180

[15] Robinson S.J., Kotwa J.D., Jeeves S.P., Himsworth C.G., Pearl D.L., Weese J.S., Lindsay L.R., Dibernardo A., Toledo N. P.L., Pickering B.S. et al. Surveillance for SARS-CoV-2 in Norway Rats (*Rattus norvegicus) from Southern Ontario*. Transbound. Emerg. Dis. 2023; 2023:1–9. 10.1155/2023/7631611 PMC12016840 40303769

[16] Goldberg A.R., Langwig K.E., Marano J., Rai P., Sharp A.K., Brown K.L., Ceci A., Kailing M.J., Briggs R., Roby C. et al. Widespread exposure to SARS-CoV-2 in wildlife communities. Nat. Commun. 2024; 15:6210. 39075057 10.1038/s41467-024-49891-w PMC11286844

[17] Rajendran M., Babbitt G.A. Persistent cross-species SARS-CoV-2 variant infectivity predicted via comparative molecular dynamics simulation. R Soc. Open Sci. 2022; 9:220600. 36340517 10.1098/rsos.220600 PMC9626255

[18] Sun B., Ni M., Liu H., Liu D. Viral intra-host evolutionary dynamics revealed via serial passage of Japanese encephalitis virus in vitro. Virus Evol. 2023; 9:veac103. 37205166 10.1093/ve/veac103 PMC10185921

[19] Messali S., Rondina A., Giovanetti M., Bonfanti C., Ciccozzi M., Caruso A., Caccuri F. Traceability of SARS-CoV-2 transmission through quasispecies analysis. J. Med. Virol. 2023; 95:e28848. 37294038 10.1002/jmv.28848

[20] Xi B., Zeng X., Chen Z., Zeng J., Huang L., Du H. SARS-CoV-2 within-host diversity of human hosts and its implications for viral immune evasion. MBio. 2023; 14:e0067923. 37273216 10.1128/mbio.00679-23 PMC10470530

[21] Armero A., Berthet N., Avarre J.-C. Intra-host diversity of SARS-Cov-2 should not be neglected: case of the state of Victoria, Australia. Viruses. 2021; 13:133. 33477885 10.3390/v13010133 PMC7833370

[22] Wertheim J.O., Wang J.C., Leelawong M., Martin D.P., Havens J.L., Chowdhury M.A., Pekar J.E., Amin H., Arroyo A., Awandare G.A. et al. Detection of SARS-CoV-2 intra-host recombination during superinfection with Alpha and Epsilon variants in New York City. Nat. Commun. 2022; 13:3645. 35752633 10.1038/s41467-022-31247-x PMC9233664

[23] Wang Y., Wang D., Zhang L., Sun W., Zhang Z., Chen W., Zhu A., Huang Y., Xiao F., Yao J. et al. Intra-host variation and evolutionary dynamics of SARS-CoV-2 populations in COVID-19 patients. Genome Med. 2021; 13:30. 33618765 10.1186/s13073-021-00847-5 PMC7898256

[24] Zhang Y., Jiang N., Qi W., Li T., Zhang Y., Wu J., Zhang H., Zhou M., Cui P., Yu T. et al. SARS-CoV-2 intra-host single-nucleotide variants associated with disease severity. Virus Evol. 2022; 8:veac106. 36505092 10.1093/ve/veac106 PMC9728387

[25] De Maio N., Gozashti L., Walker C., Corbett-Detig R. Issues with SARS-CoV-2 sequencing data. 2020; (09 September 2024, date last accessed).

[26] Roder A.E., Johnson K.E.E., Knoll M., Khalfan M., Wang B., Schultz-Cherry S., Banakis S., Kreitman A., Mederos C., Youn J.-H. et al. Optimized quantification of intra-host viral diversity in SARS-CoV-2 and influenza virus sequence data. MBio. 2023; 14:e0104623. 37389439 10.1128/mbio.01046-23 PMC10470513

[27] Hedskog C., Mild M., Jernberg J., Sherwood E., Bratt G., Leitner T., Lundeberg J., Andersson B., Albert J. Dynamics of HIV-1 quasispecies during antiviral treatment dissected using ultra-deep pyrosequencing. PLoS One. 2010; 5:e11345. 20628644 10.1371/journal.pone.0011345 PMC2898805

[28] Bull R.A., Luciani F., McElroy K., Gaudieri S., Pham S.T., Chopra A., Cameron B., Maher L., Dore G.J., White P.A. et al. Sequential bottlenecks drive viral evolution in early acute hepatitis C virus infection. PLoS Pathog. 2011; 7:e1002243. 21912520 10.1371/journal.ppat.1002243 PMC3164670

[29] Tonkin-Hill G., Martincorena I., Amato R., Lawson A. R.J., Gerstung M., Johnston I., Jackson D.K., Park N., Lensing S.V., Quail M.A. et al. Patterns of within-host genetic diversity in SARS-CoV-2. Elife. 2021; 10:e66857. 34387545 10.7554/eLife.66857 PMC8363274

[30] Novembre J., Johnson T., Bryc K., Kutalik Z., Boyko A.R., Auton A., Indap A., King K.S., Bergmann S., Nelson M.R. et al. Genes mirror geography within Europe. Nature. 2008; 456:98–101. 18758442 10.1038/nature07331 PMC2735096

[31] Platzer A. Visualization of SNPs with t-SNE. PLoS One. 2013; 8:e56883. 23457633 10.1371/journal.pone.0056883 PMC3574019

[32] Tamazian G., Komissarov A.B., Kobak D., Polyakov D., Andronov E., Nechaev S., Kryzhevich S., Porozov Y., Stepanov E. t-SNE highlights phylogenetic and temporal patterns of SARS-CoV-2 spike and nucleocapsid protein evolution. SpringerLink. 2023; 13760:255–262.

[33] Moon K.R., van Dijk D., Wang Z., Gigante S., Burkhardt D.B., Chen W.S., Yim K., van den Elzen A., Hirn M.J., Coifman R.R. et al. Visualizing structure and transitions in high-dimensional biological data. Nat. Biotechnol. 2019; 37:1482–1492. 31796933 10.1038/s41587-019-0336-3 PMC7073148

[34] Hozumi Y., Wang R., Yin C., Wei G.-W. UMAP-assisted K-means clustering of large-scale SARS-CoV-2 mutation datasets. Comput. Biol. Med. 2021; 131:104264. 33647832 10.1016/j.compbiomed.2021.104264 PMC7897976

[35] Wang B., Jiang L. Principal component analysis applications in COVID-19 genome sequence studies. Cognit. Comput. 2021; 2021:1–12. 10.1007/s12559-020-09790-w PMC7804214 33456620

[36] Mostefai F., Gamache I., N’Guessan A., Pelletier J., Huang J., Murall C.L., Pesaranghader A., Gaonac’h-Lovejoy V., Hamelin D.J., Poujol R. et al. Population genomics approaches for genetic characterization of SARS-CoV-2 lineages. Front. Med. 2022; 9:826746. 10.3389/fmed.2022.826746 PMC8899026 35265640

[37] Martin M. Cutadapt removes adapter sequences from high-throughput sequencing reads. EMBnet.journal. 2011; 17:10–12.

[38] Li H., Durbin R. Fast and accurate short read alignment with Burrows–Wheeler transform. Bioinformatics. 2009; 25:1754–1760. 19451168 10.1093/bioinformatics/btp324 PMC2705234

[39] Grubaugh N.D., Gangavarapu K., Quick J., Matteson N.L., De Jesus J.G., Main B.J., Tan A.L., Paul L.M., Brackney D.E., Grewal S. et al. An amplicon-based sequencing framework for accurately measuring intrahost virus diversity using PrimalSeq and iVar. Genome Biol. 2019; 20:8. 30621750 10.1186/s13059-018-1618-7 PMC6325816

[40] Danecek P., Bonfield J.K., Liddle J., Marshall J., Ohan V., Pollard M.O., Whitwham A., Keane T., McCarthy S.A., Davies R.M. et al. Twelve years of SAMtools and BCFtools. GigaScience. 2021; 10:giab008. 33590861 10.1093/gigascience/giab008 PMC7931819

[41] Rambaut A., Holmes E.C., O’Toole Á., Hill V., McCrone J.T., Ruis C., du Plessis L., Pybus O.G. A dynamic nomenclature proposal for SARS-CoV-2 lineages to assist genomic epidemiology. Nat. Microbiol. 2020; 5:1403–1407. 32669681 10.1038/s41564-020-0770-5 PMC7610519

[42] Guo Y., Long J., He J., Li C.-I., Cai Q., Shu X.-O., Zheng W., Li C. Exome sequencing generates high quality data in non-target regions. BMC Genomics. 2012; 13:194. 22607156 10.1186/1471-2164-13-194 PMC3416685

[43] van der Maaten L., Hinton G. Visualizing data using t-SNE. J. Mach. Learn. Res. 2008; 9:2579–2605.

[44] Pedregosa F., Varoquaux G., Gramfort A., Michel V., Thirion B., Grisel O., Blondel M., Prettenhofer P., Weiss R., Dubourg V. et al. Scikit-learn: machine learning in Python. J. Mach. Learn. Res. 2011; 12:2825–2830.

[45] Ferreira V.H., Chruscinski A., Kulasingam V., Pugh T.J., Dus T., Wouters B., Oza A., Ierullo M., Ku T., Majchrzak-Kita B. et al. Prospective observational study and serosurvey of SARS-CoV-2 infection in asymptomatic healthcare workers at a Canadian tertiary care center. PLoS One. 2021; 16:e0247258. 33592074 10.1371/journal.pone.0247258 PMC7886177

[46] Murall C.L., Fournier E., Galvez J.H., N’Guessan A., Reiling S.J., Quirion P.-O., Naderi S., Roy A.-M., Chen S.-H., Stretenowich P. et al. A small number of early introductions seeded widespread transmission of SARS-CoV-2 in Québec, Canada. Genome Med. 2021; 13:169. 34706766 10.1186/s13073-021-00986-9 PMC8550813

[47] Thielen P.M., Wohl S., Mehoke T., Ramakrishnan S., Kirsche M., Falade-Nwulia O., Trovão N.S., Ernlund A., Howser C., Sadowski N. et al. Genomic diversity of SARS-CoV-2 during early introduction into the Baltimore-Washington metropolitan area. JCI Insight. 2021; 6:e144350. 33749660 10.1172/jci.insight.144350 PMC8026189

[48] Hadfield J., Megill C., Bell S.M., Huddleston J., Potter B., Callender C., Sagulenko P., Bedford T., Neher R.A. Nextstrain: real-time tracking of pathogen evolution. Bioinformatics. 2018; 34:4121–4123. 29790939 10.1093/bioinformatics/bty407 PMC6247931

[49] Popa A., Genger J.-W., Nicholson M.D., Penz T., Schmid D., Aberle S.W., Agerer B., Lercher A., Endler L., Colaço H. et al. Genomic epidemiology of superspreading events in Austria reveals mutational dynamics and transmission properties of SARS-CoV-2. Sci. Transl. Med. 2020; 12:eabe2555. 33229462 10.1126/scitranslmed.abe2555 PMC7857414

[50] Lythgoe K.A., Hall M., Ferretti L., de Cesare M., MacIntyre-Cockett G., Trebes A., Andersson M., Otecko N., Wise E.L., Moore N. et al. SARS-CoV-2 within-host diversity and transmission. Science. 2021; 372:eabg0821. 33688063 10.1126/science.abg0821 PMC8128293

[51] Moshiri K., Mahmanzar M., Mahdavi B., Tokhanbigli S., Rahimian K., Tavakolpour S. Mutation accumulation of SARS-CoV-2 genome in North America, South America, and Oceania: Analysis of over 6.5 million sequences samples from Global Initiative on Sharing Avian Influenza Data. 2023; SSRN doi:23 November 2022, preprint: not peer reviewed 10.2139/ssrn.4282466.

[52] Fumagalli S.E., Padhiar N.H., Meyer D., Katneni U., Bar H., DiCuccio M., Komar A.A., Kimchi-Sarfaty C. Analysis of 3.5 million SARS-CoV-2 sequences reveals unique mutational trends with consistent nucleotide and codon frequencies. Virol. J. 2023; 20:31. 36812119 10.1186/s12985-023-01982-8 PMC9936480

[53] Bloom J.D., Beichman A.C., Neher R.A., Harris K. Evolution of the SARS-CoV-2 mutational spectrum. Mol. Biol. Evol. 2023; 40:msad085. 37039557 10.1093/molbev/msad085 PMC10124870

[54] Saldivar-Espinoza B., Garcia-Segura P., Novau-Ferré N., Macip G., Martínez R., Puigbò P., Cereto-Massagué A., Pujadas G., Garcia-Vallve S. The mutational landscape of SARS-CoV-2. Int. J. Mol. Sci. 2023; 24:9072. 37240420 10.3390/ijms24109072 PMC10219494

[55] Heguy A., Dimartino D., Marier C., Zappile P., Guzman E., Duerr R., Wang G., Plitnick J., Russell A., Lamson D.M., St George K. Amplification artifact in SARS-CoV-2 omicron sequences carrying P681R mutation, New York, USA. Emerg. Infect. Dis. 2022; 28:881–883. 35130474 10.3201/eid2804.220146 PMC8962901

[56] Zanini F., Brodin J., Albert J., Neher R.A. Error rates, PCR recombination, and sampling depth in HIV-1 whole genome deep sequencing. Virus Res. 2017; 239:106–114. 28039047 10.1016/j.virusres.2016.12.009

[57] McCrone J.T., Lauring A.S. Measurements of intrahost viral diversity are extremely sensitive to systematic errors in variant calling. J. Virol. 2016; 90:6884–6895. 27194763 10.1128/JVI.00667-16 PMC4944299

[58] Illingworth C. J.R., Roy S., Beale M.A., Tutill H., Williams R., Breuer J. On the effective depth of viral sequence data. Virus Evol. 2017; 3:vex030. 29250429 10.1093/ve/vex030 PMC5724399

[59] Dinis J.M., Florek K.R., Fatola O.O., Moncla L.H., Mutschler J.P., Charlier O.K., Meece J.K., Belongia E.A., Friedrich T.C. Deep sequencing reveals potential antigenic variants at low frequencies in influenza A virus-infected humans. J. Virol. 2016; 90:3355–3365. 26739054 10.1128/JVI.03248-15 PMC4794676

[60] N’Guessan A., Kailasam S., Mostefai F., Poujol R., Grenier J.-C., Ismailova N., Contini P., De Palma R., Haber C., Stadler V., Bourque G., Hussin J.G., Shapiro B.J., Fritz J.H., Piccirillo C.A. Selection for immune evasion in SARS-CoV-2 revealed by high-resolution epitope mapping and sequence analysis. iScience. 2023; 26:107394. 37599818 10.1016/j.isci.2023.107394 PMC10433132

[61] McElroy K., Zagordi O., Bull R., Luciani F., Beerenwinkel N. Accurate single nucleotide variant detection in viral populations by combining probabilistic clustering with a statistical test of strand bias. BMC Genomics. 2013; 14:501. 23879730 10.1186/1471-2164-14-501 PMC3848937

[62] Mostefai F., Grenier J.-C., Poujol R., Hussin J. SARS-CoV-2 intra-host mutational landscape: a curated dataset of iSNVs. Mendeley Data. 2024; 10.17632/8nvgtrkzdm.2.

[63] Chen Z., Azman A.S., Chen X., Zou J., Tian Y., Sun R., Xu X., Wu Y., Lu W., Ge S. et al. Global landscape of SARS-CoV-2 genomic surveillance and data sharing. Nat. Genet. 2022; 54:499–507. 35347305 10.1038/s41588-022-01033-y PMC9005350

[64] Smith E.A., Libuit K.G., Kapsak C.J., Scribner M.R., Wright S.M., Bell J., Morales C., Crumpler M., Messenger S., Hacker J.K. et al. Pathogen genomics in public health laboratories: successes, challenges, and lessons learned from California’s SARS-CoV-2 Whole-Genome Sequencing Initiative, California COVIDNet. Microb Genom. 2023; 9:mgen001027. 37267020 10.1099/mgen.0.001027 PMC10327507

[65] Tapinos A., Constantinides B., Phan M. V.T., Kouchaki S., Cotten M., Robertson D.L. The utility of data transformation for alignment, de novo assembly and classification of short read virus sequences. Viruses. 2019; 11:394. 31035503 10.3390/v11050394 PMC6563281

[66] Paradis E. Reduced multidimensional scaling. Comput. Stat. 2022; 37:91–105.

[67] Karim M.R., Islam T., Shajalal M., Beyan O., Lange C., Cochez M., Rebholz-Schuhmann D., Decker S. Explainable AI for bioinformatics: methods, tools and applications. Briefings Bioinf. 2023; 24:bbad236. 10.1093/bib/bbad236 37478371

[68] Muyas F., Sauer C.M., Valle-Inclán J.E., Li R., Rahbari R., Mitchell T.J., Hormoz S., Cortés-Ciriano I. De novo detection of somatic mutations in high-throughput single-cell profiling data sets. Nat. Biotechnol. 2023; 42:758–767. 37414936 10.1038/s41587-023-01863-z PMC11098751

[69] Short P.J., McRae J.F., Gallone G., Sifrim A., Won H., Geschwind D.H., Wright C.F., Firth H.V., FitzPatrick D.R., Barrett J.C. et al. De novo mutations in regulatory elements in neurodevelopmental disorders. Nature. 2018; 555:611–616. 29562236 10.1038/nature25983 PMC5912909

[70] Keegan K.P., Glass E.M., Meyer F. MG-RAST, a metagenomics service for analysis of microbial community structure and function. Methods Mol Biol. 2016; 1399:207–233. 26791506 10.1007/978-1-4939-3369-3_13

[71] Vatteroni M.L., Capria A.-L., Spezia P.G., Frateschi S., Pistello M. Co-infection with SARS-CoV-2 omicron BA.1 and BA.2 subvariants in a non-vaccinated woman. Lancet Microbe. 2022; 3:e478. 35623374 10.1016/S2666-5247(22)00119-7 PMC9129255

[72] Rockett R.J., Draper J., Gall M., Sim E.M., Arnott A., Agius J.E., Johnson-Mackinnon J., Fong W., Martinez E., Drew A.P. et al. Co-infection with SARS-CoV-2 Omicron and Delta variants revealed by genomic surveillance. Nat. Commun. 2022; 13:2745. 35585202 10.1038/s41467-022-30518-x PMC9117272

[73] Fox E.J., Reid-Bayliss K.S., Emond M.J., Loeb L.A. Accuracy of next generation sequencing platforms. Next Gener. Seq. Appl. 2014; 1:1000106. 25699289 10.4172/jngsa.1000106 PMC4331009

[74] Cook R., Brown N., Rihtman B., Michniewski S., Redgwell T., Clokie M., Stekel D.J., Chen Y., Scanlan D.J., Hobman J.L., Nelson A., Jones M.A., Smith D., Millard A. The long and short of it: benchmarking viromics using Illumina, Nanopore and PacBio sequencing technologies. Microb Genom. 2024; 10:001198. 38376377 10.1099/mgen.0.001198 PMC10926689

[75] Fournelle D., Mostefai F., Brunet-Ratnasingham E., Poujol R., Grenier J.-C., Gálvez J.H., Pagliuzza A., Levade I., Moreira S., Benlarbi M. et al. Intra-host evolution analyses in an immunosuppressed patient supports SARS-CoV-2 viral reservoir hypothesis. Viruses. 2024; 16:342. 38543708 10.3390/v16030342 PMC10974702

[76] Caron E., Kovalchik K., Hamelin D., Kubiniok P., Bourdin B., Mostefai F., Poujol R., Paré B., Simpson S., Sidney J. et al. Integrating machine learning-enhanced immunopeptidomics and SARS-CoV-2 population-scale analyses unveils novel antigenic features for Next-generation COVID-19 vaccines. 2024; Research Square doi:12 February 2024, preprint: not peer reviewed https://www.researchsquare.com/article/rs-3914861/v1.

[77] Bradley C.C., Wang C., Gordon A. J.E., Wen A.X., Luna P.N., Cooke M.B., Kohrn B.F., Kennedy S.R., Avadhanula V., Piedra P.A. et al. Targeted accurate RNA consensus sequencing (tARC-seq) reveals mechanisms of replication error affecting SARS-CoV-2 divergence. Nat. Microbiol. 2024; 9:1382–1392. 38649410 10.1038/s41564-024-01655-4 PMC11384275

[78] Crits-Christoph A., Kantor R.S., Olm M.R., Whitney O.N., Al-Shayeb B., Lou Y.C., Flamholz A., Kennedy L.C., Greenwald H., Hinkle A. et al. Genome sequencing of sewage detects regionally prevalent SARS-CoV-2 variants. mBio. 2021; 12:e02703-20. 33468686 10.1128/mBio.02703-20 PMC7845645

