# Supplementary material for: Refining SARS-CoV-2 intra-host variation by leveraging large-scale sequencing data
Source: NAR Genom Bioinform. 2024 Nov 12;6(4):lqae145. doi: 10.1093/nargab/lqae145 (PMC11555433; doi:10.1093/nargab/lqae145)
Supplement: lqae145_Supplemental_File [file lqae145_supplemental_file.pdf]

Supplemental Tables and Figures

Table S1. iSNV Count, Library Count, and Mutational Load

	iSNV Count	Library Count	Mutational Load
Total iSNVs	11,635,231	128,443	91
Consensus iSNVs	3,634,563	128,323	28
De novo iSNVs	8,000,668	128,352	62
$S > 1\%$ filtered *de novo* iSNVs	6,508,783	127,941	51
Masked *de novo* iSNVs	5,805,486	125,382	46
$AAF > 5\%$ filtered *de novo* iSNVs	468,651	73,729	6
Non-Outlier libraries *de novo* iSNVs	296,437	72,470	4

Table S2. Per Country Sequencing Libraries' Counts Before and After Coverage Filters.

	Countries	Before Filters (C_l)	After Filters (B_l)
Africa	Angola	519	266
	Cameroon	210	102
	Ethiopia	125	47
	Malawi	428	175
	Mozambique	287	152
	South Africa	3,662	2,527
	Zimbabwe	507	342
	Other ($n < 100$)	181	117
Asia	China	115	106
	India	437	350
	Israel	609	538
	Lebanon	367	275
	Pakistan	227	149
	Other ($n < 100$)	100	92
Europe	Austria	543	542
	Estonia	5,817	4,704
	Finland	5,331	4,394
	Greece	2,614	2,231
	Italy	452	323
	Norway	3,376	1,139
	Portugal	11,700	10,502
	Slovakia	5,982	5,553
	Switzerland	379	375
	United Kingdom	74,557	69,710
	Other ($n < 100$)	129	101
North America	Canada	625	588
	USA	20,880	16,300
	Other ($n < 100$)	91	82
Oceania	Australia	6,837	6,421
	Northern Mariana Islands	23	22
South America	Brazil	417	186
	Other ($n < 100$)	10	9
**Total**		**147,537**	**128,420**

Table S3. Per Sequencing Center Libraries' Counts Before and After Coverage Filters.

**Sequencing Center**	Before Filters (C_l)	After Filters (B_l)
Wellcome Sanger Institute	69,676	65,316
National Institute Of Health DR. Ricardo Jorge	11,700	10,502
Doherty Institute	6,699	6,306
CDC-OAMD	6,431	5,040
Ravi Kant	5,331	4,394
Kwazulu-Natal Sequencing Platform	4,711	2,827
Comenius University in Bratislava	4,648	4,458
University Of Tartu, Estonia	4,104	3,351
Norwegian Institute of Public Health (NIPH)	3,376	1,139
INAB Institute, Certh	2,614	2,231
BROAD, GCID	2,334	1,948
Chan Zuckerberg Biohub	1,971	1,850
Quadram Institute Bioscience	1,859	1,257
UPHL ID	1,853	1,062
Institute of Biomedicine and Translational Medicine	1,713	1,353
Wales Specialist Virology Centre	1,507	1,485
Chan Zuckerberg Biohub	1,364	1,229
Public Health Authority of the Slovak Republic	1,349	1,106
TX-SARS-COV-2	1,324	710
Public Health England (Colindale)	1,142	1,123
DCLS-NGS	1,062	492
California Department of Public Health	982	900
Liverpool Clinical Laboratories	825	741
CanCOGeN CPHLN	612	579
Tel Aviv University	609	538
NYC SARS-COV-2	562	493
CeMM	543	542
New Mexico Department of Health Scientific Laboratory	539	503
Colorado Department of Public Health and Environment	509	463
Gujarat Biotechnology Research Centre	436	349
West of Scotland Specialist Virology Centre, NHSGG	362	362
University Hospital of Basel	336	336
Delaware Public Health Lab	327	299
University of Kwazulu-Natal	277	180
Network for Genomic Surveillance in South Africa	275	274
CDC-PDD	253	222
Utah Public Health lab	233	169
Kwazulu-Natal Research and Sequencing Platform	229	137
UMIGS	223	209
University of Verona	220	188
SEARCH	211	191
Hospital Israelita Albert Einstein	208	0
SciLifeLab Stockholm	164	133
Institute of Clinical Pathology and Medical Research	138	135
LNCC	119	110
Ruijin Hospital, Shanghai Jiao Tong University of Medicine	112	103
Centers for Disease Control and Prevention	100	95

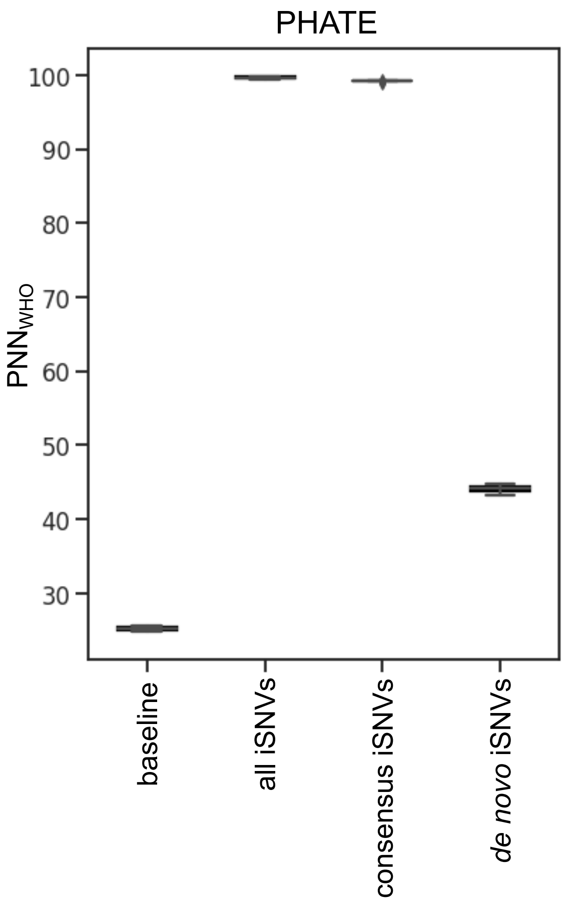

Fig. S1. Unveiling WHO Lineage Patterns in SARS-CoV-2 iSNVs with PHATE Visualizations and PNN_{WHO} Metric. Boxplots show the distribution of the mean percentage of nearest neighbours (PNN_{WHO}) from the same WHO lineage annotation across libraries for each PHATE (**A**) visualization across the ten replicates from the sub-sampling controlled experiment (see Method section 2.5). Before computing PNN_{WHO}, PHATE visualizations were generated on matrices containing a consistent sampling of 4,000 libraries from each of Alpha, Delta, Omicron, and Beta WHO annotated lineages. For PHATE, the first boxplot represents the expected PNN_{WHO} values by chance, followed by all iSNVs, consensus only iSNVs, and *de novo* only iSNVs. The number of nearest neighbours used in this experiment is k=40.

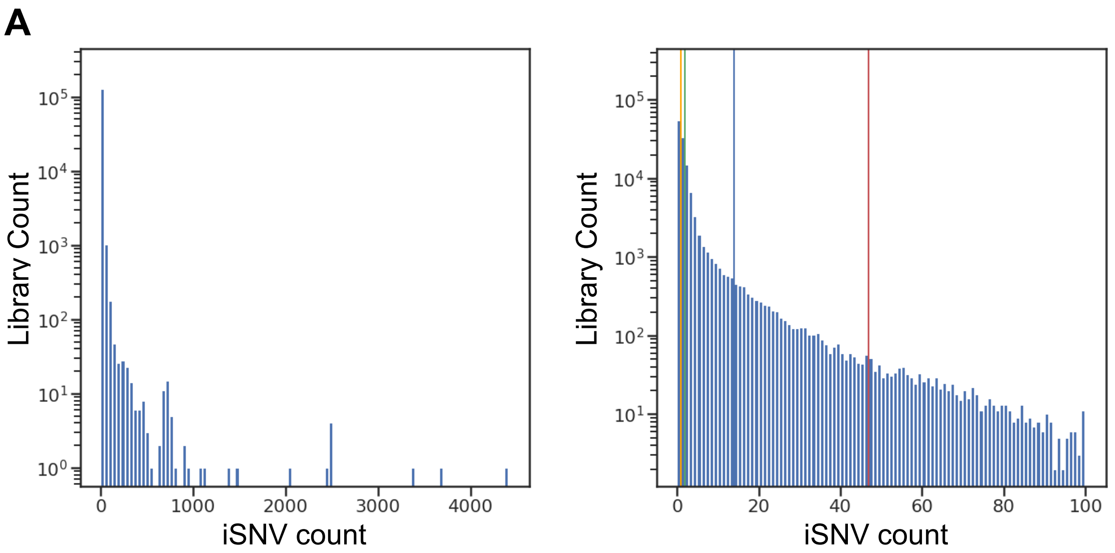

B

	Threshold	Outlier Libraries	PHATE (baseline)	t-SNE (baseline)
All libraries	N/A	N/A	36.23% (27.68%)	38.18% (27.68%)
99th percentile	44 iSNVs/l	1,270	33.26% (26.29%)	36.11% (26.28%)
95th percentile	14 iSNVs/l	6,156	33.32% (27.18%)	36.92% (27.24%)
75th percentile	2 iSNVs/l	24,185	32.70% (28.74%)	35.93% (29.69%)
50th percentile	1 iSNV/l	37,934	32.33% (29.00%)	33.03% (29.05%)

Fig. S2. Analysis of Libraries' Mutational Load, which is the number of iSNVs per library. **A** The left panel shows the mutational load distribution across all libraries highlighting the variability in the number of iSNVs per library. The right panel provides a zoomed-in view of this distribution, focusing on libraries with up to 100 iSNVs. This view includes vertical lines to delineate various distribution percentiles. **B** A table summarizing the relationship between different outlier detection thresholds and their impact on library clustering structure on the PHATE visualizations. The table shows thresholds defined by the number of iSNVs per library, ranging from the 99th percentile (44 iSNVs/library) to the 50th percentile (1 iSNV/library). For each threshold, the table indicates the number of libraries classified as outliers and the corresponding percentage of nearest neighbours from the same WHO lineage (PNN_{WHO}), alongside the expected by chance PNN_{WHO} value.

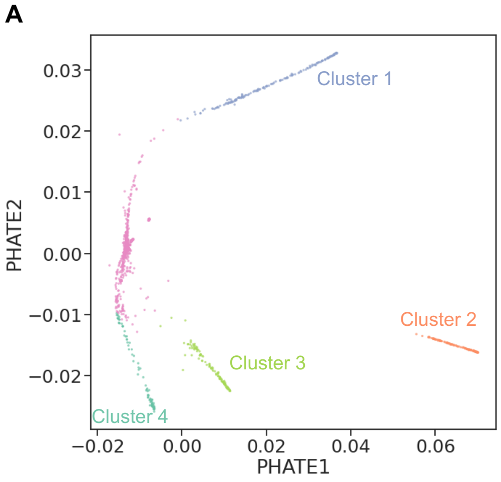

B

Cluster	Number of Libraries	Sequencing Center (Count from Center)	% of Center's Libraries in our dataset	Sample Counts by Month/Year
1	164	Doherty Institute (159)	2.65%	March 2020: 77, August 2020: 54, July 2020: 26, April 2020: 5, May 2020: 1,
2	104	Scilifelab Stockholm (104)	78.20%	December 2020: 1 January 2021: 98, February 2021: 6
3	109	Kwazulu-Natal Platform (106)	3.86%	January 2021: 20, February 2021: 25, March 2021: 43, April 2021: 21
4	81	Ravi Kant Sequencing (75)	1.84%	May 2021: 42, February 2021: 15, June 2021: 10, April 2021: 5, March 2021: 5, January 2021: 3

Fig. S3. **A**: PHATE on the top 1% outlier libraries with the most iSNV count. The clusters on this PHATE representation were defined using K-means applied to the PHATE object. **B**: Table representing per cluster library information.

Supplemental Information

Details on Downloading SARS-CoV-2 Genomic Libraries from NCBI

A total of 147,537 SARS-CoV-2 Illumina amplicon paired-end sequencing reads were downloaded from NCBI, as follows: 51,837 Illumina SARS-CoV-2 sequencing libraries were downloaded from the NCBI database on June 4th, 2021, and another 95,700 Illumina sequencing libraries were downloaded on February 12th, 2022. January and February 2020 were severely underrepresented compared to the other months (Figure 2A). Most downloaded sequences originated from Europe, constituting 75% of the dataset. Among the European sequences, 63% were obtained from the Wellcome Sanger Institute sequencing center, UK (Table S3), indicating their significant contribution to the global sequencing efforts. Furthermore, a notable number of downloaded libraries came from The Doherty sequencing center, Australia, between January and October 2020 (16% of the total libraries Table S3) as they led the sequencing effort during that time in that region. Additionally, the dataset was enriched with samples sequenced by North American sequencing centers, accounting for 15% of the downloaded sequences (Tables S2 and S3). The underrepresentation of samples from January and February 2020 reflects a limitation in the available data during the initial stages of the pandemic. However, despite the initial disparities in data collection, which reflect the current practical challenges faced by the scientific community [63], this dataset remains highly informative, successfully capturing the global diversity of SARS-CoV-2 throughout the later months of 2020 and extending into 2021.

Out of the total libraries downloaded, 134,879 had a mean coverage C above 100, and a total of 138,723 libraries had a breadth of coverage B above 10,000, meaning that at least 10,000 genomic positions were covered at a depth of 100X or higher (Figure 2). The intersection of both filters allowed us to keep 128,423 high-quality sequencing libraries for further analysis. The distributions of the breadth of coverage and mean depth show heterogeneity in the coverage of the downloaded sequencing libraries. We also note the grouping of some sequencing centers (e.g. Welcome Sanger Institute in red) and not others (e.g. the CDC's Office of Advanced Molecular Detection - CDC-OAMD), displaying a heterogeneity across sequencing centers and within sequencing centers. Because we downloaded a representative sampling of the available data on the NCBI database, this coverage distribution likely represents the coverage heterogeneity of the available data on NCBI.

Strand Coverage Across the Genome

We evaluated the variation in strand coverage along the genome in our dataset using the Forward Strand Ratio (FSR), which revealed a highly unbalanced distribution across the virus sequence (Figure 2C). Only 31% of the viral genome in our dataset has a balanced coverage from the forward and reverse read strands. Specifically, 40% of the genome is covered by the plus strand, which is the number of genomic positions of the genome with an average forward strand ratio above 90%. In contrast, 29% of the genome is covered by the minus strand, with an average minus strand ratio above 90%. Thus, strand bias statistics in SARS-CoV-2 genomes need to consider strand coverage when evaluating if a *de novo* iSNVs is a stand bias artifact, which motivates the development of our strand bias likelihood metric S.

Recurrent Strand Bias Artifacts

To better characterize strand bias artifacts, we analyzed a total of 1,491,885 intra-host single nucleotide variants (iSNVs) identified as potential strand bias artifacts, with a likelihood of no strand bias below 1% ($S < 0.01$). We first examined their alternative allele frequency (AAF) distribution. The AAF distribution of these excluded iSNVs does not differ significantly from that of the other iSNVs, suggesting that strand bias artifacts can happen across a spectrum of intra-host frequencies. This confirms that filtering based solely on AAF is insufficient to eliminate strand bias artifacts.

Several genomic positions were found to be recurrent within these putative strand bias artifacts. We computed the expected number of libraries with strand bias artifacts at a given position, which has a mean of 4 and a 99th percentile of 68 libraries. We identified 486 genomic positions that have a strand bias artifact reported in more than 68 libraries, labelling them as recurrent strand bias artifacts, which we masked in our analyses across all libraries. To ensure the robustness of iSNV analyses and to prevent the inclusion of recurrent spurious iSNVs, we recommend evaluating and possibly masking these genomic positions in future SARS-CoV-2 intra-host studies.

Sub-sampling experiments to balance WHO variants

In our dataset, Alpha and Delta are overrepresented compared to other SARS-CoV-2 variants, which may cause biases in the analysis results since unbalanced sampling can influence cluster formation and PNN_{WHO} values (see, for example, Figure 3A, which distinctly marks Alpha and Delta as dominant clusters). To address this, we conducted controlled sub-sampling experiments, selecting 1,000 libraries each from the Alpha, Beta, Delta, and Omicron variants (see Method section 2.5), aiming to mitigate variant sampling bias on PNN_{WHO} values in the PHATE representation of iSNV subsets. We evaluated the clustering by WHO lineage across three iSNV sets: unfiltered raw iSNVs, consensus iSNVs, and *de novo* iSNVs (Figure S1). The raw and consensus iSNV datasets show high PNN_{WHO} values, indicating a strong lineage-specific signature, primarily driven by frequent lineage-defining mutations, even when samples per WHO variant are balanced. Conversely, *de novo* iSNVs exhibit lower PNN_{WHO} values, indicating a subtler lineage-based structure but still above baseline, underscoring the lineage-specific biological significance of emerging mutations. These controlled subsampling experiments thus replicate our main findings with the full dataset (Figure 4). Therefore, the lineage-specific signatures observed in our study are not a result of the uneven sampling of WHO variants.

t-SNE Results Are Comparable to PHATE

In this section, we present results from t-SNE (t-Distributed Stochastic Neighbor Embedding) analysis of SARS-CoV-2 genomic data, complementing the PHATE results found in the result section (see results section 3). The method t-SNE is a machine learning algorithm used for dimensionality reduction, offering an alternative approach to PHATE.

The t-SNE representation of the 128,423 high-quality sequencing libraries reveals distinct clusters by WHO lineage for both raw and consensus iSNV subsets, consistent with PHATE's findings. For raw iSNVs, the Proportion of Nearest Neighbors (PNN_{WHO}) for t-SNE is 99.43%, closely aligned with PHATE's 98.39%. Similarly, for consensus iSNVs, t-SNE's PNN_{WHO} of 99.05% parallels PHATE's 99.37%, highlighting both methods' consistent ability to identify lineage-specific mutations across the iSNV sets. Conversely, *de novo* iSNVs (representing emerging mutations within the host) show less pronounced lineage-specific than consensus iSNVs clustering in t-SNE representation, with a PNN_{WHO} value of 59.37%. This suggests a deviation from the strong lineage alignment observed in raw and consensus iSNVs, indicating that while *de novo* iSNVs still correlate with lineage structure more than baseline, the association is less direct. The structure observed in *de novo* iSNVs through t-SNE complements PHATE's analysis, demonstrating consistent underlying data patterns regardless of the representation method used.

Using the 8,000,668 unfiltered *de novo* iSNVs, both t-SNE and PHATE visualizations revealed significant sequencing center batch effects, with t-SNE showing slightly higher PNN_{SC} values (66.50%) compared to PHATE (62.31%). This indicates that both dimensionality reduction techniques captured the influence of sequencing center-specific artifacts within the *de novo* iSNV dataset. Efforts to refine the dataset and mitigate these artifacts involved applying thresholds on the S and AAF metrics. These measures effectively reduced sequencing center-specific artifacts, as evidenced by decreased PNN_{SC} values in both visualization methods after applying the filters, with the t-SNE value (38.18%) slightly higher than PHATE (36.23%). Applying the filters effectively reduced sequencing center-specific artifacts, as evidenced by decreased PNN_{SC} values in both representation methods.

Similarly to PHATE, we also computed the PNN_{SC} values in t-SNE representation after sequentially removing the top 1%, 5%, and 25% of the libraries with the most iSNV counts (Figure S2B). As opposed to PHATE, the PNN_{SC} value of t-SNE did not drastically decrease after the removal of the top 1% of our outliers. However, the PNN_{SC} values for both t-SNE and PHATE only met after the exclusion of more libraries down to only keeping libraries with one iSNV (Figure S2B, 50th percentile), underlining the stronger impact of outlier libraries on t-SNE compared to PHATE.

Similar to the approach used with PHATE, we calculated the PNN_{SC} values for t-SNE after removing the top 1%, 5%, and 25% of libraries based on iSNV counts (Figure S2B). Unlike PHATE, the PNN_{SC} for t-SNE did not significantly decrease with the removal of the top 1% of libraries. Both t-SNE and PHATE PNN_{SC} values converged after removing more libraries, ultimately comparable for their PNN_{SC} values only when retaining those with a single iSNV (Figure S2B, 50th percentile). This indicates that t-SNE is more susceptible to bias from outlier libraries compared to PHATE.

This overall consistency between dimensionality reduction methods serves as compelling evidence that the data's underlying structure is method-independent, suggesting that both methods could be reliably applied to similar datasets to help inform future pre-processing strategies in viral genomics. This alignment helps validate our pre-processing strategies in viral genomics, demonstrating the robustness of our observations and the general applicability of these techniques to analyze viral genomic data.
